# Systemic Coagulation Derangement as an Early Sign of Oxygenator Failure in Veno-Venous Extracorporeal Membrane Oxygenation (VV ECMO) Without Anticoagulation

**DOI:** 10.3390/reports7040097

**Published:** 2024-11-12

**Authors:** Konstanty Szułdrzyński, Miłosz Jankowski, Magdalena Fleming

**Affiliations:** 1 Department of Anesthesiology and Intensive Care, National Institute of Medicine of the Ministry of Interior and Administration, 02-507 Warsaw, Poland; 2 Department of Anesthesiology and Intensive Care, Professor Adam Gruca Teaching Hospital, Postgraduate Medical Education Centre, 05-400 Otwock, Poland

**Keywords:** VV ECMO, thrombosis, DIC, coagulopathy, oxygenator failure

## Abstract

**Background and Clinical Significance**: Veno-venous extracorporeal membrane oxygenation (VV ECMO) has become a widely accepted supportive treatment for severe acute respiratory distress syndrome (ARDS) in intensive care units (ICUs). Although it has gained popularity, some of its aspects, including optimal anticoagulation management and the best means of monitoring hemostasis, remain unresolved. Thrombosis and bleeding are still important complications of ECMO. **Case Presentation**: A 44-year-old male patient, with no underlying conditions, was diagnosed with severe acute respiratory distress syndrome (ARDS) due to AH1N1 influenza. He presented severe hypoxemia despite the use of mechanical ventilation, neuromuscular blocking agent infusion and prone position. VV ECMO was used, and coagulation was stopped on ECLS day 6 due to severe pulmonary hemorrhage. The systemic hemostatic disorders found in this patient were difficult to differentiate from disseminated intravascular coagulation (DIC) or sepsis-induced coagulopathy (SIC), improved transiently after circuit exchange, and resolved only after discontinuation of ECMO. The patient was discharged fully conscious and cooperative, with no apparent neurological deficit. **Conclusions**: Systemic hemostatic abnormalities may precede oxygenator failure and mimic DIC or SIC. Timely oxygenator exchange may therefore be considered. However, it is a high-risk procedure, especially in fully ECLS-dependent patients.

## 1. Introduction and Clinical Significance

Since the encouraging results of the CESAR trial and its successful application in the A/H1N1 pandemic, veno-venous extracorporeal membrane oxygenation (VV ECMO) has become a widely accepted supportive treatment for severe ARDS in intensive care units (ICUs) [1]. Although it has gained popularity, some of its aspects, including optimal anticoagulation management and the best means of monitoring hemostasis, remain unresolved. Thrombosis and bleeding are still important complications of ECMO. Contact of blood with the artificial surfaces of the ECMO circuit, as well as shear stress created by the high velocity of the blood flow, leads to platelet activation and enhanced fibrin production [2]. Furthermore, the inflammation—usually enhanced in ECMO patients—also excites coagulation. Simultaneously, the reliability of the ECMO circuit is of the utmost importance for the survival of patients completely dependent on extracorporeal life support (ECLS). Therefore, anticoagulation remains the cornerstone of treatment with ECLS.

Clinical significance: In this case report, we highlight the decision-making process regarding the timing of circuit exchange due to coagulopathy reminiscent of disseminated intravascular coagulopathy (DIC) and the management of anticoagulation and bleeding in a fully ECLS-dependent critically ill patient with AH1/N1 pneumonia and respiratory failure.

## 2. Case Presentation

A 44-year-old male patient, with no underlying conditions, was transferred to the tertiary academic hospital from a district hospital due to severe ARDS with an unknown cause. Five days prior to the initial hospital admission, the patient was treated with amoxicillin and azithromycin for suspected bacterial pneumonia in an outpatient clinic. The patient presented to the emergency department (ED) of a district hospital due to breathlessness and was admitted to the local internal medicine ward. Several hours later, due to rapid deterioration, he was transferred to the intensive care unit (ICU) of the district hospital, where he was immediately intubated and mechanical ventilation was initiated. Due to the further deterioration of his condition despite the intensification of treatment, the patient was transferred to the academic hospital on day 2 of his hospital stay.

His body mass index (BMI) was 27.4 kg/m^2^, and his body surface area (BSA) was 2.2 m^2^. The patient was admitted already in a prone position, intubated and mechanically ventilated (MV) for 24 h. He was sedated with propofol, fentanyl, and midazolam infusions. Furthermore, cisatracurium was administered as a neuromuscular blockade. Mechanical ventilation was maintained in assist control/pressure control mode (AC/PC) with driving pressure (dP) of 14 cm H_2_O, total positive end-expiratory pressure (tPEEP) 14 cm H_2_O, peak pressure 32 cm H_2_O, plateau pressure 28 cm H_2_O, FiO_2_ 1.0, inspiratory time 0.8 s, and respiratory rate of 24 breaths/minute. Continuous infusion of norepinephrine was necessary due to hypotension, with an invasive arterial blood pressure of 80/50 mmHg, aimed at maintaining a mean arterial pressure (MAP) > 65 mmHg. His renal and liver function was normal. The initial arterial blood gas revealed a pH of 7.303, PaO_2_ of 10,759 kPa (80.7 mmHg), P/F ratio 80.7, PaCO_2_ of 8265 kPa (62 mmHg), and HCO_3_^−^ of 26 mmol/L. The severity of the patient’s condition was estimated according to the following scales: APACHE II: 20; SOFA score: 12; ECMOnet score 7; Murray score: 4. Chest X-ray revealed massive, confluent, bilateral opacities. Meropenem, levofloxacin and fluconazole were continued (second day of treatment) as an empirical treatment. Empiric oseltamivir was initiated for suspected influenza based on clinical judgement despite the negative test performed in the referring hospital.

**Figure 1:**
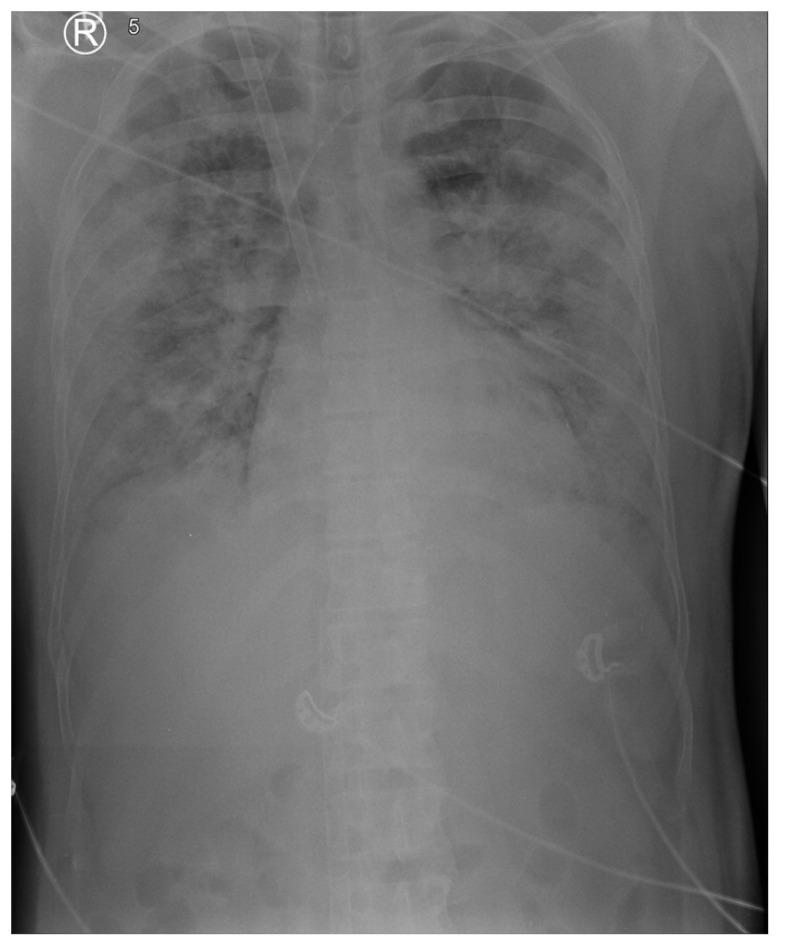
Chest X-ray on ECLS day 1. Chest X-ray revealed massive, confluent, bilateral opacities.

Over the 3 h following admission, the patient’s oxygenation worsened. After a short period in the supine position due to the insertion of a left subclavian central vein catheter (CVC), the patient was placed in a prone position again, with minimal benefit. The recruitment maneuver (BILEVEL: PEEP High 40 cm H_2_O, PEEP Low 20 cm H_2_O, I:E = 5:1 for 1 min) yielded no lung recruitment or improvement in oxygenation. As the hypoxemia persisted with a PaO_2_/FiO_2_ ratio below 80 despite optimized ventilation with optimal individual PEEP determined (decremental PEEP staircase titration according to the best compliance), cisatracurium infusion and prone positioning, VV ECMO support was instituted (ECLS day 1/MV day 2) using a Cardiohelp console with an HLS 7.0 set (both from Maquet, Rastatt, Germany). Using the ultrasound-guided Seldinger technique, a drainage cannula was inserted into the right femoral vein (venous, French 23, 55 cm) and a return cannula into the right jugular vein (arterial, French 19, 15 cm). A follow-up chest radiograph and bedside ultrasound were performed to confirm the cannula positioning (Figure 1).

The initial ECMO blood flow was 5.0 L/min at 3000 RPM, and the sweep gas flow was 6.0 L/min. Mechanical ventilation was modified to AC/PC with a dP of 10 cm H_2_O, PEEP 12 cm H_2_O, FiO_2_ 0.4, tidal volume (TV)~3 mL/kg PBW and a respiratory rate of 5/min. According to the local ECMO anticoagulation protocol, a bolus of heparin (2000 units) was given intravenously, then unfractionated heparin (UFH) was administered at an initial dose of 700 IU/h in continuous infusion aiming to maintain an APTT of 45–50 s (initial APTT × 1.5 − 2). On ECLS day 4, a positive rtPCR test for A/H1N1 was obtained from bronchoalveolar lavage (performed on the admission day), so treatment with oseltamivir was continued. On ECLS day 5, continuous intravenous infusion of cisatracurium was tapered off, but it was reinstated after several hours due to the respiratory distress and worsening oxygenation. On ECLS day 6, a massive intrapulmonary bleeding occurred, leading to a nearly complete occlusion of the airway (APTT 48 s). The tidal volume during pressure-controlled ventilation dropped to 100 mL. Heparin was withheld immediately. The blood flow through the ECMO circuit was increased to 6.0 L/min (4200 RPM) and the FiO_2_ of the ventilator was set at 1.0 to maintain SpO_2_ > 80% (above 6l/min, suctioning of the drainage cannula occurred despite optimal fluid resuscitation according to the bedside ultrasound).

Flexible bronchofiberoscopy was performed to evacuate fragments of blood clots. A cryoprobe was not available in our department. The endotracheal tube was changed due to the full occlusion by clots. Improvements in oxygenation and ventilation were achieved after the bronchoscopy, the ECMO parameters were returned to previous settings, but the tidal volume during mechanical ventilation remained low (150 mL). On consecutive days, bleeding diminished significantly; however, coagulation tests revealed symptoms of activated clotting. The D-dimer levels were rising, and fibrinogen declined gradually. The APTT levels remained stable at around 35 s. On ECLS day 9, mechanical ventilation was no longer possible due to the airway thrombosis. Attempts to remove the thrombi from the trachea and bronchial tree proved to be futile despite prolonged flexible bronchofiberoscopy performed daily (ECLS days 9–15). The ECMO remained fully functional with stable values of the pressure gradient across the oxygenator and unaltered oxygen transfer. However, on ECLS day 16, UFH infusion was reestablished at 500 IU/h, aiming at an APTT of 40 s to preserve ECMO function in the face of ineffective mechanical ventilation and complete ECLS-dependence. A decline in D-dimers with a moderate increase in the fibrinogen levels were noted. The day after (ECLS day 17), recurrent intrapulmonary bleeding occurred, leading to the final cessation of anticoagulation. Over the following days, the D-dimer levels were constantly increasing, and fibrinogen was declining, with more pronounced systemic coagulation impairment and minor bleeding at the ECMO cannulation sites, catheter insertion sites and from the oropharyngeal cavity. A chest CT scan revealed no symptoms of pulmonary embolism, and the inflammatory parameters remained consistently low. Deep vein thrombosis in the lower extremities was ruled out by the bedside ultrasound.

**Figure 2:**
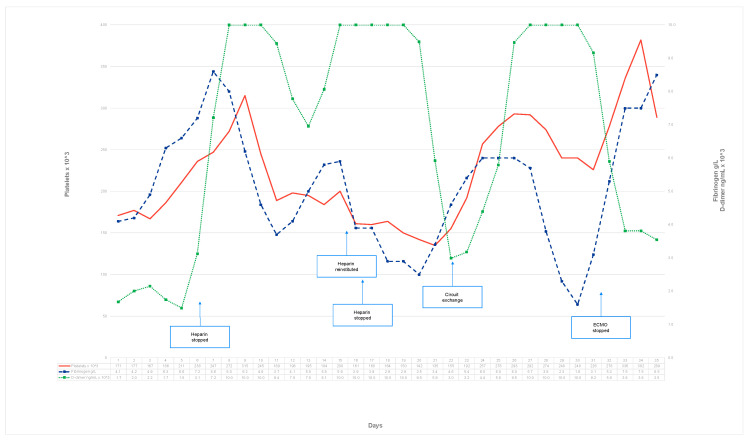
Timeline of the coagulation parameters and interventions during ECLS.

Despite the unaltered oxygenator gas exchange function—PaO_2_ behind the oxygenator consistently >200 mm Hg—and lack of increase in the oxygenator hemodynamic resistance (constant delta P), an ECMO circuit was replaced on ECLS day 22 in an attempt to correct the systemic coagulation derangement. After the circuit exchange, a rapid improvement in the hemostatic parameters was noted, with a decrease in the D-dimer levels and a fibrinogen increase. However, these parameters returned to the previous trend over the next few days. Simultaneously, the pulmonary bleeding stopped, and the thrombi were finally removed from the airway. The lungs were reaerated and their function gradually recovered. On ECLS day 24, the endotracheal tube was replaced with a size 9 due to the persistent leakage of a size 8.5. Tracheostomy was not performed during the ECMO treatment. ECMO was discontinued on ECLS day 32. The coagulation parameters returned to normal 2 days after the ECMO decannulation (Figure 2). No signs of deep vein thrombosis were detected via ultrasound. The patient was extubated on MV day 39 and discharged to the district hospital 68 days after admission, fully conscious and cooperative, with no apparent neurological deficit. In the two-year follow-up, he was able to return to work.

## 3. Discussion

ECMO is a supportive treatment for severe ARDS that is of growing popularity. It has proved to be at least as effective as mechanical ventilation alone and the results in A/H1N1 influenza-associated ARDS were particularly promising [1]. So far, therapeutic anticoagulation remains the mainstay of clinical practice in terms of ECMO. Thrombotic complications of ECMO are frequent and often life-threatening. Furthermore, oxygenator failure due to thrombosis is one of the most gruesome problems with the procedure [3]. Protein and cellular deposits on the oxygenator membrane surface impede gas exchange long before an overt thrombosis occurs [4]. Therefore, cautious monitoring of the anticoagulation levels, precise dosing of anticoagulants and timely circuit exchange are of the utmost importance. However, oxygenator replacement is associated with a risk of serious complications, including severe hypoxemia and cardiac arrest.

Importantly, hemostatic derangements are often present in ECMO patients for reasons independent of the extracorporeal therapy itself, such as inflammation and infection, liver failure, bleeding and subsequent deficiency of clotting factors or deep vein thrombosis (DVT) frequently occurring in ECMO patients [5]. Moreover, a low platelet count may be a result of inflammation, platelet activation and destruction within the circuit or a side effect of medication. DIC and sepsis-induced coagulopathy (SIC) may also occur in ECMO patients, and their diagnosis is particularly challenging. All the guidelines, including the recent edition from the International Society of Thrombosis and Hemostasis, base the recognition of DIC on a dynamic assessment of a number of parameters, including decreased fibrinogen levels, increased levels of fibrin degradation products, increased prothrombin time and decreased platelet count [6]. Unfortunately, in ECMO patients, most of these parameters may be altered due to reasons other than DIC itself, predominantly degradation of the ECMO circuit and inflammation. Recently, a new set of criteria were proposed for recognition of SIC, reflecting the need to adopt the DIC diagnostic tools to the characteristics of septic patients [7]. Nonetheless, the diagnosis of DIC or SIC is even more difficult when anticoagulation must be discontinued for massive bleeding in ECMO patients.

Several markers of activated coagulation have been proposed as indicators of imminent ECMO circuit failure due to extracorporeal clotting. Most often, elevated D-dimers, thrombin–antithrombin complexes (TAT) and soluble fibrin were associated with this event [2,8]. However, despite some data regarding the predictive value of soluble fibrin, there is no clear evidence that any of these markers provide reliable differentiation between intra- and extracorporeal coagulation, allowing the definitive distinction between DIC or SIC and circuit clotting.

The case we present highlights that systemic hemostatic abnormalities may precede oxygenator failure and mimic DIC or SIC. Timely oxygenator replacement may therefore improve coagulation parameters. However, prolonged ECMO support without any anticoagulation may also be dangerous, leading to the clotting of the ECMO circuit, DVT and pulmonary embolism or even peripheral embolism when a patent foramen ovale is present, which happens in up to 20% of patients with severe ARDS [9]. Recent findings point to the role of a hypercoagulable state and attenuated fibrinolysis in severe cases of ARDS associated with COVID-19 [10].

Meanwhile, the continuous anticoagulation warranted by the ECMO circuit efficacy may itself be a reason for life-threatening complications, including intracranial hemorrhage and bleeding from the oropharyngeal cavity, cannulation sites, gastrointestinal and urinary tracts. In our case, anticoagulation caused severe pulmonary bleeding, rendering the patient entirely dependent on the extracorporeal support. Cessation of anticoagulation was therefore necessary; however, it led to recurrent oxygenator thrombosis—a problem finally resolved by weaning from ECMO. Simultaneously, the complete dependence of the patient on the ECLS makes the decision to stop anticoagulation very difficult. Exchange of the extracorporeal circuit despite the lack of visible thrombosis or increased hemodynamic resistance of the oxygenator significantly improved the coagulation parameters, most likely because the thrombus forming on the membrane lung consumed platelets and fibrinogen.

Based on the presented case, we hypothesize that cessation of anticoagulation during ECMO may lead to secondary hemorrhagic diathesis due to the depletion of clotting factors used in the process of clotting inside the oxygenator, rendering the diagnosis of the primary cause of hemostatic derangement very difficult. Moreover, systemic coagulation disorders—especially severe thrombocytopenia, decreased levels of fibrinogen and elevated D-dimers—may indicate extracorporeal circuit thrombosis and warrant its replacement.

## 4. Conclusions

Systemic hemostatic abnormalities may precede oxygenator failure and mimic DIC or SIC. Timely oxygenator exchange may therefore be considered despite adequate gas exchange and satisfactory resistance. However, it is a high-risk procedure, especially in fully ECLS-dependent patients.

## Data Availability

The original data presented in the study are included in the article, further inquiries can be directed to the corresponding author.

## References

[1] Combes A., Schmidt M., Hodgson C.L., Fan E., Ferguson N.D., Fraser J.F., Jaber S., Pesenti A., Ranieri M., Rowan K. (2020). Extracorporeal life support for adults with acute respiratory distress syndrome. Intensive Care Med..

[2] Hoshino K., Muranishi K., Kawano Y., Hatomoto H., Yamasaki S., Nakamura Y., Ishikura H. (2020). Soluble fibrin is a useful marker for predicting extracorporeal membrane oxygenation circuit exchange because of circuit clots. J. Artif. Organs.

[3] Lubnow M., Philipp A., Foltan M., Bull Enger T., Lunz D., Bein T., Haneya A., Schmid C., Riegger G., Müller T. (2014). Technical complications during venovenous extracorporeal membrane oxygenation and their relevance predicting a system-exchange–retrospective analysis of 265 cases. PLoS ONE.

[4] Lehle K., Philipp A., Gleich O., Holzamer A., Müller T., Bein T., Schmid C. (2008). Efficiency in extracorporeal membrane oxygenation—Cellular deposits on polymethypentene membranes increase resistance to blood flow and reduce gas exchange capacity. ASAIO J..

[5] Fisser C., Reichenbächer C., Müller T., Schneckenpointner R., Malfertheiner M.V., Philipp A., Foltan M., Lunz D., Zeman F., Lubnow M. (2019). Incidence and risk factors for cannula-related venous thrombosis after venovenous extracorporeal membrane oxygenation in adult patients with acute respiratory failure. Crit. Care Med..

[6] Wada H., Thachil J., Di Nisio M., Mathew P., Kurosawa S., Gando S., Kim H.K., Nielsen J.D., Dempfle C.E., Levi M. (2013). Guidance for diagnosis and treatment of DIC from harmonization of the recommendations from three guidelines. J. Thromb. Haemost..

[7] Iba T., Levy J.H., Levy J.H., Warkentin T.E., Thachil J., Van Der Poll T., Levi M., Scientific and Standardization Committee on DIC, and the Scientific and Standardization Committee on Perioperative and Critical Care of the International Society on Thrombosis and Haemostasis (2019). Diagnosis and management of sepsis-induced coagulopathy and disseminated intravascular coagulation. J. Thromb. Haemost..

[8] Lubnow M., Philipp A., Dornia C., Schroll S., Bein T., Creutzenberg M., Diez C., Schmid C., Pfeifer M., Riegger G. (2014). D-dimers as an early marker for oxygenator exchange in extracorporeal membrane oxygenation. J. Crit. Care.

[9] Dessap A.M., Boissier F., Leon R., Carreira S., Campo F.R., Lemaire F., Brochard L. (2010). Prevalence and prognosis of shunting across patent foramen ovale during acute respiratory distress syndrome. Crit. Care Med..

[10] SB M.J., Chacko B., Selvarajan S., Peter J.V., Geevar T., Dave R.G., Georgy J.T., Zachariah A., George T., Sathyendra S. (2024). Biomarkers of coagulation, endothelial, platelet function, and fibrinolysis in patients with COVID-19: A prospective study. Sci. Rep..

